# International time trends in sudden unexpected infant death, 1969–2012

**DOI:** 10.1186/s12887-020-02271-x

**Published:** 2020-08-11

**Authors:** Jacqueline Müller-Nordhorn, Alice Schneider, Ulrike Grittner, Konrad Neumann, Thomas Keil, Stefan N. Willich, Sylvia Binting

**Affiliations:** 1 Institute for Social Medicine, Epidemiology and Health Economics, Charité - Universitätsmedizin Berlin, Luisenstr. 57, 10117 Berlin, Germany; 2 Bavarian Cancer Registry, Bavarian Health and Food Safety Authority, Nuremberg, Germany; 3 Institute of Biometry and Clinical Epidemiology, Charité - Universitätsmedizin Berlin, Berlin, Germany; 4 Berlin Institute of Health, Berlin, Germany; 5 Institute for Clinical Epidemiology and Biometry, University of Wuerzburg, Wuerzburg, Germany; 6 Institute for Health Resort Medicine and Health Promotion, Bavarian Health and Food Safety Authority, Bad Kissingen, Germany

**Keywords:** Sudden unexpected infant death, Sudden infant death syndrome, Time trends, Country-clusters

## Abstract

**Background:**

Sudden unexpected infant death (SUID) - including sudden infant death syndrome (SIDS) - continues to be a major contributor to infant mortality worldwide. Our objective was to analyse time trends and to identify country-clusters.

**Methods:**

The National Statistical Offices of 52 countries provided the number of deaths and live births (1969–2012). We calculated infant mortality rates per 1000 live births for SUID, SIDS, and all-cause mortality. Overall, 29 countries provided sufficient data for time course analyses of SUID. To sensitively model change over time, we smoothed the curves of mortality rates (1980–2010). We performed a hierarchical cluster analysis to identify clusters of time trends for SUID and SIDS, including all-cause infant mortality.

**Results:**

All-cause infant mortality declined from 28.5 to 4.8 per 1000 live births (mean 12.4; 95% confidence interval 12.0–12.9) between 1969 and 2012. The cluster analysis revealed four country-clusters. Clusters 1 and 2 mostly contained countries showing the typical peak of SUID mortality during the 1980s. Cluster 1 had higher SUID mortality compared to cluster 2. All-cause infant mortality was low in both clusters but higher in cluster 1 compared to cluster 2. Clusters 3 and 4 had low rates of SUID without a peak during the 1980s. Cluster 3 had the highest all-cause infant mortality of all clusters. Cluster 4 had an intermediate all-cause infant mortality. The time trends of SUID and SIDS mortality were similar.

**Conclusions:**

The country-specific time trends in SUID varied considerably. The identification of country-clusters may promote research into how changes in sleep position, smoking, immunisation, or other factors are related to our findings.

## Background

Mortality from sudden infant death syndrome (SIDS) is still a major contributor to mortality in the first year of life worldwide [1]. In many Western countries, including Western Europe, Australia, Canada, New Zealand, and the United States, mortality from SIDS peaked in the 1980s and decreased during the 1990s [2–6]. In other countries, such as Japan, SIDS mortality was low during the 1980s and subsequently increased [7, 8]. The decrease in SIDS mortality in Western countries has been attributed mainly to the ‘Back to Sleep’ campaigns promoting the supine sleep position [2, 3, 9]. In the United States, for example, the National Infant Sleep Position Study showed an increase in the supine sleep position from 17% in 1993 to 72% in 2007 [10].

Gilbert et al. assessed the time frame for ‘Back-to-Sleep’ campaigns in various countries in a systematic review [9]. The campaigns often coincided with reductions in SIDS. The International Child Care Practice Study, however, found large variations in infant sleep position between countries [11]. For example, the prevalence of the supine sleep position was 33% in Denmark compared to 89% in Japan in the late 1990s. While infant sleep position and other risk factors act as triggering factors, the underlying cause(s) for SIDS are still unknown [12]. The success of the ‘Back-to-Sleep’ campaigns might have covered concurrent changes in other factors at the population level. Known risk factors for SIDS other than the prone or side sleep position include bed sharing, soft bedding, mothers’ smoking and alcohol use, overheating, and lack of immunisation [4, 12, 13].

Determining regional time trends for SIDS mortality and identifying clusters of time courses might instigate new research into the aetiology of SIDS. As the coding of SIDS varies between countries, the broader category of sudden unexpected infant death may be more appropriate for international comparisons [14]. The term sudden unexpected death in infancy (SUDI) is often used interchangeably with SUID as an umbrella term for unexplained infant deaths [15]. During recent years, diagnostic shifts have been reported from SIDS to other diagnoses [16, 17]. Sudden unexpected infant death (SUID) typically includes SIDS, accidental suffocation and strangulation in bed, or other ill-defined or unspecified causes of death [13]. When comparing SUID mortality, all-cause infant mortality needs to be taken into account as well. In countries with high all-cause infant mortality, vulnerable infants might die earlier from other causes. Therefore, the objective of the present study was to identify country-clusters with similar time trends in SUID and SIDS as well as in all-cause infant mortality in an international comparison.

## Methods

### Study design

The present study is a comparison of historical time trends in SUID, SIDS, and all-cause infant mortality between countries across the globe (1969–2012). Infant deaths were defined as deaths in children during the first year of life. We obtained data from the National Statistical Offices of the respective countries. In the case of missing data, we checked the World Health Organization (WHO) Mortality Database and included additional data if available [18]. Diagnoses were used according to the International Classification of Diseases (ICD) systems [19]. Our primary diagnosis of interest was SUID. The diagnosis of SUID commonly includes SIDS (ICD-10, R95), accidental suffocation and strangulation in bed (ICD10, W75), or other ill-defined or unspecified causes of mortality (ICD-10, R99) [14, 16]. We used the broader category ill-defined and unknown causes of mortality (ICD-10, R96–99), as international comparisons have shown differences in the use of individual codes of diagnoses between countries [14]. For example, a high percentage of SUID was coded as other sudden death, cause unknown (ICD-10, R96) in Japan [14]. Codes of diagnoses used for SUID and related diagnoses might differ both between and within countries over time. During the period of interest, the ICD systems changed [19]. We used the following ICD systems: the 8th revision (ICD-8), the 9th revision (ICD-9), and the 10th revision (ICD-10) (Table 1). The years in which ICD systems changed differed between countries.

**Table 1:** International Classification of Diseases (ICD) codes for sudden infant death syndrome, related diagnoses and all causes of death

Codes of diseases	ICD-8	ICD-9	ICD-10
Sudden infant death syndrome	795	798.0	R95
Accidental suffocation and strangulation in bed	E913.0	E913.0	W75
Ill-defined and unknown causes of mortality	796	798.1–798.9, 799	R96-R99
All causes of death	000-E999	001-E999	A00-Y89

A number of countries used other classification systems, such as the 09 N – ICD 9th revision, Special List of causes (tabulation list) (countries of the former Union of Soviet Socialist Republics, USSR), the 09A/09B – List ICD 9th revision, Standard Basic Tabulation (Croatia, Greece, Iceland, Japan, New Zealand), or the Finnish Classification of Diseases 1987. The German Democratic Republic (GDR), which existed until 1990, used a special version of the ICD for the coding of deaths. For the latest years of our study, all countries - apart from Greece - had adopted the ICD-10 codes. The causes of death in Greece were coded with ICD-9 until 2013.

### Regions and countries

For the classification of regions, we used geographic units that were adapted from the Global Burden of Disease Study [1]. We included the following regions and countries of interest in our study, focusing on Europe, with selected countries from other regions of the world for comparisons:

1. Western Europe (Austria, Belgium, Cyprus, Denmark, East Germany, England & Wales, Finland, France, Greece, Iceland, Ireland, Italy, Luxembourg, Malta, Netherlands, Northern Ireland, Norway, Portugal, Scotland, Spain, Sweden, Switzerland, West Germany) excluding Andorra, Liechtenstein, Monaco, and San Marino due to the small population sizes (≤90,000 inhabitants). We did not provide a total estimate for the United Kingdom due to the differential use of ICD systems. Similarly, we included East and West Germany separately due to differences in coding and classification systems used over time.
2. Central Europe (Albania, Bulgaria, Croatia, Czech Republic, Hungary, Kosovo, Republic of Macedonia, Poland, Romania, Serbia, Slovakia, Slovenia).
3. Eastern Europe (Belarus, Estonia, Latvia, Lithuania, Republic of Moldova, the Russian Federation, Ukraine).
4. Selected countries from other regions: high-income North America (Canada, USA), Australia (Australia, New Zealand), high-income Asia Pacific (Japan), Southern Latin America (Chile, Uruguay), Central Latin America (Costa Rica, Mexico), and North Africa and Middle East (Turkey).

### Time period and data collection

We used all data on infant mortality with the respective codes of diagnoses for the time period from 1969 to 2012 for the descriptive analyses [20]. For the cluster analyses of time trends, we restricted the time period to the years from 1980 to 2010 due to the large amount of missing data for the earlier and later years. We included 29 countries for the cluster analyses of time trends in SUID mortality and 27 countries for SIDS mortality, respectively.

The format (paper-based, digital) and degree of segregation of the data varied considerably between countries. Some countries only provided aggregated data for the ICD category symptoms, signs and abnormal clinical and laboratory findings, not elsewhere classified (ICD-10, R00-R99) but not separately for the diagnoses SIDS (ICD-10, R95), accidental suffocation and strangulation in bed (ICD-10, W75), or ill-defined and unknown causes of mortality (ICD-10, R96–99).

### Statistical analyses

We calculated infant mortality rates per 1000 by dividing the number of infant deaths with the respective diagnoses by the number of live births multiplied by 1000. For the descriptive analyses, we divided mortality rates from SUID mortality into quintiles over 3-year periods. We calculated the distribution over these quintiles with 1980–1982 as the reference years for both previous and subsequent years. We used maps to display the distribution of SUID mortality rates graphically for the years 1970, 1980, 1990, 2000, and 2010. To create the maps, we used the software EASYMAP 11.0 SP 6 (@2018 Luttum+ Tappert DV-Beratung GmbH, Bonn, www.lutumtappert.de).

Mortality rates from SUID can be affected by all-cause mortality rates. Therefore, we examined the time trends of mortality rates from SUID, SIDS, and death from all-causes. The time series of mortality rates were smoothed before further analysis using restricted cubic splines with six nodes. Smoothing data removed noise from the data and allowed us to sensitively model changes over time. We performed hierarchical cluster analyses to identify similar time courses of SUID and SIDS across countries. Countries were clustered for SUID and all-cause mortality as well as for SIDS and all-cause infant mortality. We used the values of the smoothed SUID, SIDS, and all-cause infant mortality curves from 1980 to 2010 for the cluster analyses. In total, 62 variables were the basis for each of the two cluster analyses. Because the higher levels of all-cause infant mortality would give all-cause infant mortality a greater weight in the cluster analyses, we calculated Manhattan distance matrices for SUID and all-cause mortality separately and averaged both distance matrices. Thus, we were able to ensure equal weight of SUID and all-cause mortality in the cluster analysis. The distance matrix for clustering SIDS mortality was calculated accordingly. Finally, the hierarchical cluster algorithm used Ward’s minimum variance method. We calculated country-specific maxima over time based on the smoothed curves for the mortality rates from SUID and SIDS. For the calculation of the restricted cubic splines, we used the R package “rms”. The cluster analyses were carried out using the hclust function from the statistical software R 3.3.2 (R Foundation for Statistical Computing, Vienna).

## Results

**Fig. 1:**
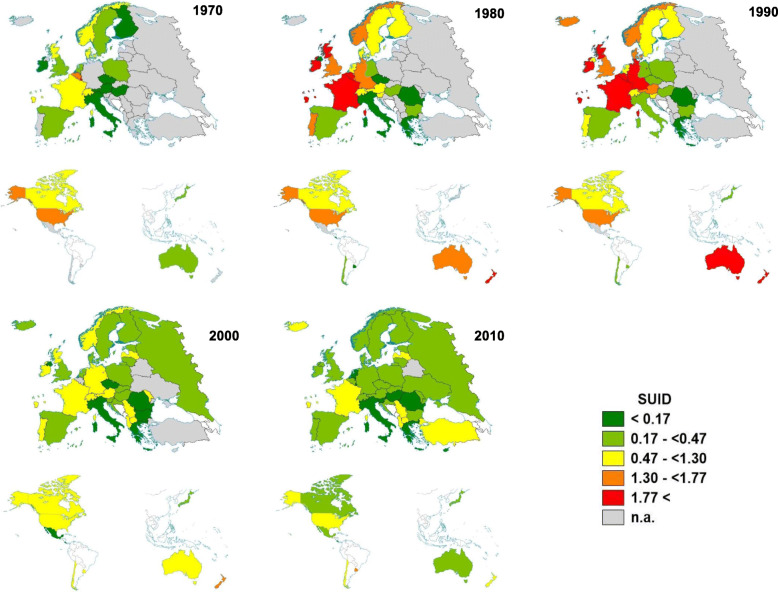
Regional distribution of infant mortality rates from sudden unexpected infant death (SUID) per 1000 live births in 10-year intervals; top: Europe, bottom left: Americas, bottom right: Australia, Japan, New Zealand

In total, 52 countries provided data on infant mortality. All-cause infant mortality decreased from an average of 28.5 per 1000 live births in 1969 to 4.8 in 2012 (mean mortality rate over all years: 12.4; 95% confidence interval 12.0–12.9). While all-cause infant mortality rates were available for all countries from 1969 to 2012, the completeness of available mortality data to calculate SUID mortality was initially low; however, it improved during the time period of interest. Data on SUID were available for 22 countries in 1970, 32 in 1980, 35 in 1990, 45 in 2000, and 49 in 2010. Mortality from SUID declined in most regions. Figure 1 shows the geographical distribution of SUID mortality rates for the years 1970, 1980, 1990, 2000, and 2010.

**Fig. 2:**
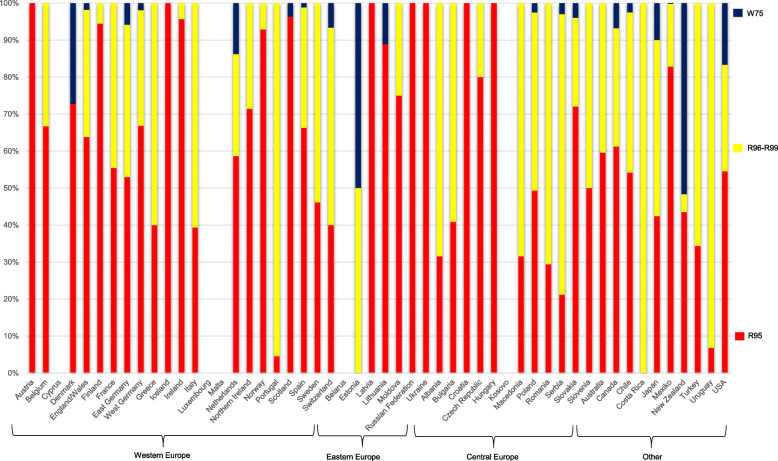
Distribution of sudden unexpected infant death by its component diagnoses, according to country (2010); SIDS (ICD-10, R95), ill-defined and unknown causes of mortality (ICD-10, R96–99), accidental suffocation and strangulation in bed (ICD10, W75)

Differences existed in the use of codes of diagnoses between countries. The percentage of SIDS mortality among SUID mortality ranged between 30 and 40% from 1969 to 1976, rose steadily to 83% in 1994, and declined again, ranging between 60 and 70% from 1995 onwards. In 1970, for example, Austria, Finland and France did not code any cases of SUID as SIDS, whereas the Czech Republic, Luxembourg, and Poland coded all SUID cases as SIDS. Differences persisted over time. In 2010, only a low percentage of SUID cases was coded as SIDS in Costa Rica and Estonia (both 0%) and Portugal (5%), whereas Austria, Croatia, Hungary, Iceland, Latvia, the Russian Federation, and Ukraine coded all SUID as SIDS. Figure 2 shows the distribution of the respective diagnoses among SUID mortality rates according to country (year 2010). The distribution of diagnoses over time for the preceding decades (1980, 1990, 2000) is shown in the Additional file 1.

**Fig. 3:**
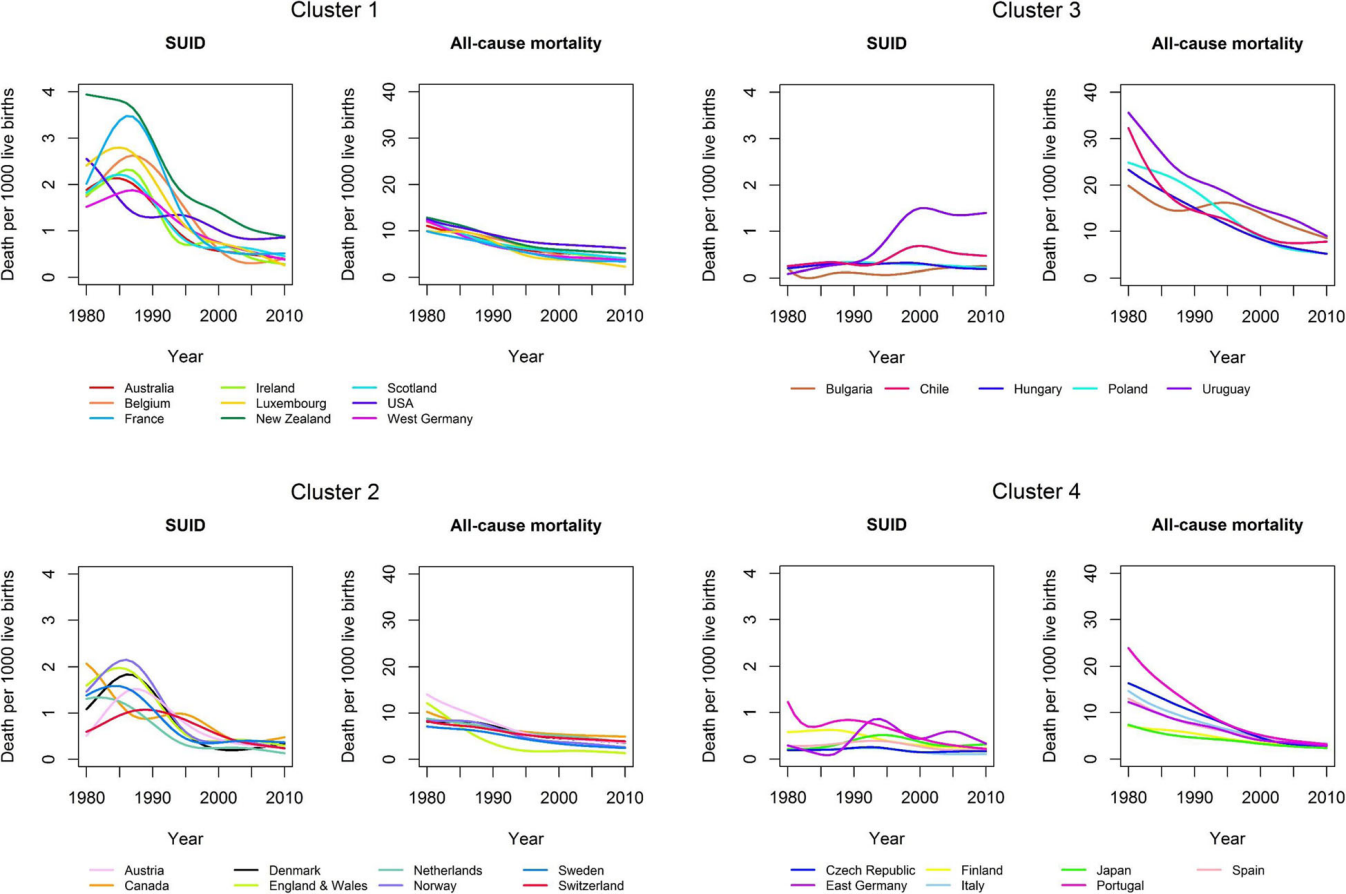
Country-clusters of sudden unexpected infant death (SUID) and all-cause infant mortality (1980–2010)

Time trends for SUID mortality rates from 29 countries were grouped into four clusters (Fig. 3, Table 2). Table 2 shows the maxima of SUID mortality per country-cluster, based on smoothed curves. The main difference between cluster 1 and cluster 2 countries with regard to SUID were the lower SUID and all-cause mortality rates in cluster 2. The maximum of SUID rates was 3.9 per 1000 live births (New Zealand) with the lowest value of 1.9 for West Germany in cluster 1, the maximum of SUID rates in cluster 2 was 2.2 (Norway) with the lowest value of 1.1 for Switzerland. With regard to the dynamic, SUID rates decreased from around 2.1 in 1990 to 1.1 in 1995 in cluster 1, while they decreased from around 1.3 to 0.6 during the same time period in cluster 2. Cluster 1 included mainly countries from Western Europe (Belgium, France, Ireland, Luxembourg, Scotland, West Germany) as well as Australia, New Zealand and the USA. Cluster 2 included Austria, Canada, Denmark, England & Wales, Netherlands, Norway, Sweden, Switzerland. In cluster 3 (Bulgaria, Chile, Hungary, Poland, Uruguay), mortality rates from SUID were low, while all-cause infant mortality was approximately 2-fold higher compared to clusters 1 and 2. Mortality rates from SUID remained below 1 (except for Uruguay in 2001). Cluster 4 (Czech Republic, East Germany, Finland, Italy, Japan, Portugal, Spain), similarly, had low mortality rates from SUID. All-cause infant mortality rates were lower in cluster 4 compared to cluster 3.

**Table 2:** Maximum of sudden unexpected infant death (SUID) per 1000 live births per country-cluster (based on smoothed curves)

	Maximum of SUID	Year of maximum
**Cluster 1**		
Australia	2.14	1984
Belgium	2.63	1987
France	3.48	1986
Ireland	2.32	1986
Luxembourg	2.80	1985
New Zealand	3.94	1980
Scotland	2.22	1985
USA	2.56	1980
West Germany	1.88	1987
**Cluster 2**		
Austria	1.52	1987
Canada	2.07	1980
Denmark	1.83	1986
England & Wales	1.98	1985
Netherlands	1.34	1982
Norway	2.15	1986
Sweden	1.59	1984
Switzerland	1.07	1989
**Cluster 3**		
Bulgaria	0.25	2010
Chile	0.69	2000
Hungary	0.32	1998
Poland	0.33	1990
Uruguay	1.50	2001
**Cluster 4**		
Czech Republic	0.26	1992
East Germany	0.86	1994
Finland	0.63	1986
Italy	0.24	1992
Japan	0.52	1994
Portugal	1.23	1980
Spain	0.39	1993

**Fig. 4:**
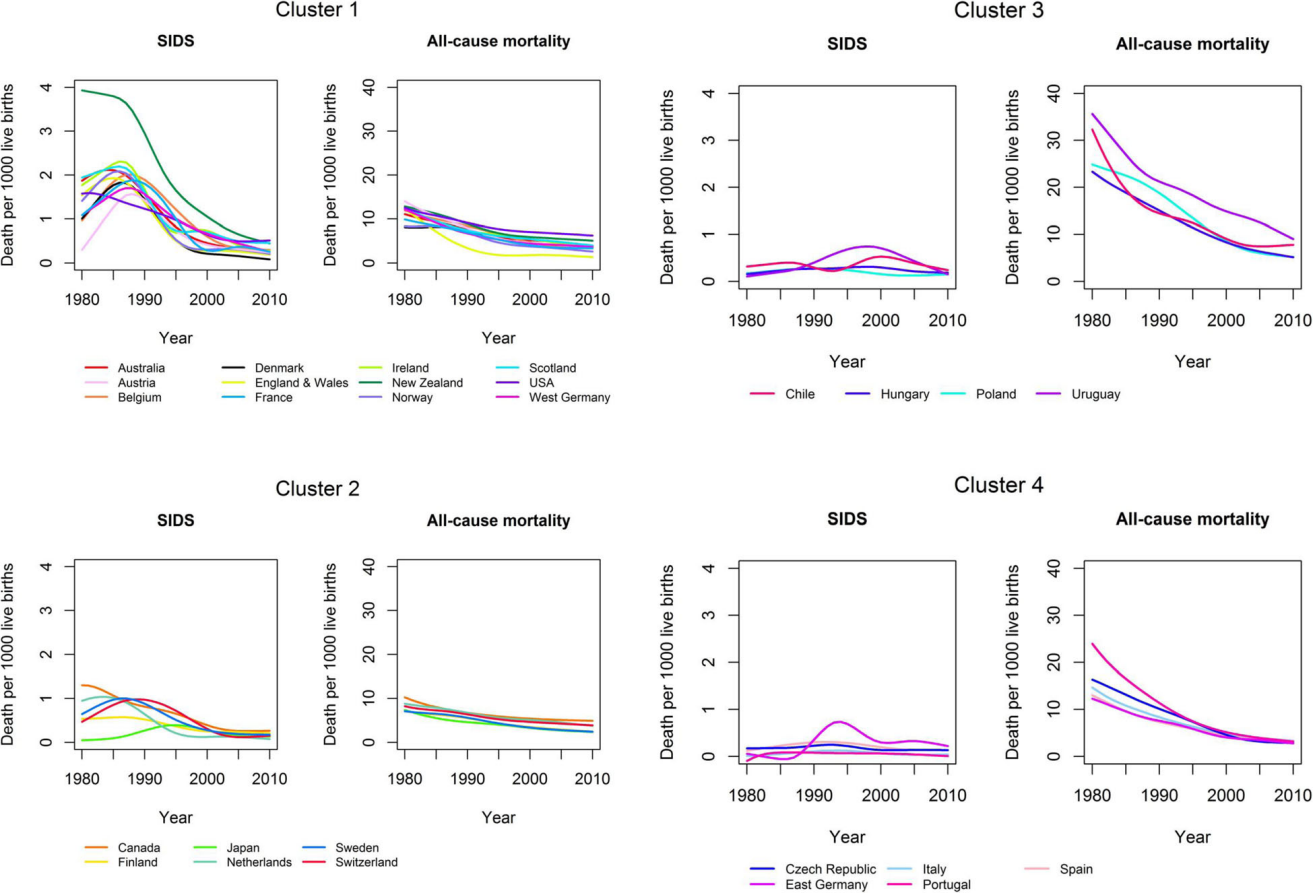
Country-clusters of sudden infant death syndrome (SIDS) and all-cause infant mortality (1980–2010)

Time trends for SIDS mortality rates from 27 countries were grouped into four clusters (Fig. 4, Table 3). Table 3 shows the maxima of SIDS mortality per country-cluster, based on smoothed curves. The differences between clusters 1 and 2 in the cluster analysis of SIDS were similar to those of SUID (Table 2, Table 3). Most of the countries were in the same clusters (Cluster 1: Australia, Belgium, France, Ireland, New Zealand, Scotland, USA, West Germany; Cluster 2: Canada, Sweden, Switzerland, the Netherlands) in both analyses. Some of the countries could only be analysed with regard to one of the outcomes SIDS or SUID (Luxemburg, Finland, Japan). In four countries, the cluster allocation was different for SIDS compared to SUID: Austria, Denmark, England & Wales and Norway. All four were in cluster 1 for SIDS but in cluster 2 for SUID. For these countries, rates of SIDS and SUID were almost identical and rates of SIDS were higher than in countries of cluster 2 (SIDS). When analysing SUID rates in these countries, they were lower than in other countries of cluster 1. Cluster 1 included 12 countries predominantly from Western Europe as well as Australia, New Zealand and the USA. A peak of SIDS mortality was reported between 1980 and 1988 (range: 1.6–3.9). Following the peak in mortality, SIDS mortality rates decreased until 2010. New Zealand had the highest SIDS mortality of all countries. All-cause infant mortality in cluster 1 was between 7 and 15 and decreased continuously from 1980 onwards. Countries of cluster 2 (Canada, Finland, Japan, Netherlands, Sweden, Switzerland) showed similar trends in SIDS mortality compared to cluster 1 but at a lower level. A maximum of SIDS mortality was reported between 1980 and 1995 (range: 0.4–1.3). Clusters 3 (Chile, Hungary, Poland, Uruguay) and 4 (Czech Republic, East Germany, Italy, Portugal, Spain) had low SIDS mortality rates (below 1) but differed with regard to all-cause infant mortality. Cluster 3 had the highest all-cause infant mortality of all clusters, while cluster 4 had intermediate infant all-mortality.

**Table 3:** Maximum of sudden infant death syndrome (SIDS) per 1000 live births per country-cluster (based on smoothed curves)

	Maximum of SIDS	Year of maximum
**Cluster 1**		
Australia	2.12	1984
Austria	1.57	1988
Belgium	2.01	1988
Denmark	1.83	1986
England & Wales	1.93	1985
France	1.88	1988
Ireland	2.31	1986
New Zealand	3.93	1980
Norway	2.09	1986
Scotland	2.20	1986
USA	1.59	1981
West Germany	1.70	1987
**Cluster 2**		
Canada	1.30	1980
Finland	0.57	1986
Japan	0.39	1995
Netherlands	1.04	1983
Sweden	1.00	1987
Switzerland	0.98	1989
**Cluster 3**		
Chile	0.53	2000
Hungary	0.31	1998
Poland	0.28	1990
Uruguay	0.75	1998
**Cluster 4**		
Czech Republic	0.25	1993
East Germany	0.74	1994
Italy	0.13	1993
Portugal	0.09	1986
Spain	0.31	1992

## Discussion

All-cause infant mortality as well as SUID and SIDS mortality declined in most countries. The cluster analyses yielded four country-clusters for both SUID and SIDS. Two of the clusters showed the typical peak in SUID and SIDS mortality observed during the 1980s, mainly in countries from Western Europe as well as Australia, Canada, New Zealand, and the United States. These clusters had a low all-cause infant mortality but differed with regard to their levels of SUID and SIDS mortality. The remaining two clusters had high and intermediate all-cause infant mortality, with low mortality from SUID and SIDS. These clusters predominantly included countries from Central Europe as well as some countries from the Mediterranean region.

Most studies comparing international time trends have focused on SIDS but not SUID mortality [1, 2]. Coding practices for SIDS and SIDS-related diagnoses, however, vary considerably between countries [14]. In Japan, for example, only approximately 40% of SUID cases are coded as SIDS [14]. Whereas the R96 diagnosis (other sudden death, cause unknown) is predominantly used as an alternative to SIDS in Japan, other countries, such as Canada, England & Wales, Germany, or the United States, are more likely to use the code R99 (other ill-defined and unspecified causes of mortality) or W75 (accidental suffocation and strangulation in bed). Comparing time trends in SUID thus allows for a more robust comparison between countries and over time. We also included all-cause infant mortality in our cluster analyses. The low SUID mortality found in the clusters with high and intermediate all-cause infant mortality may at least partially be due to vulnerable children dying earlier from other causes. In particular, mortality from perinatal conditions was increased in the countries with high and intermediate all-cause mortality, as well as mortality from infections in countries with high all-cause mortality [18].

The initial increase and subsequent decrease in SIDS mortality in many countries has been attributed to changes in infant sleep position [3, 5, 9]. Campaigns promoting the supine sleep position started in most countries during the early 1990s [9]. While the change in infant sleep position is a major factor associated with reducing SIDS mortality, other changes in potential risk factors at the population level have received less attention. For example, immunisation against pertussis decreased in a number of countries during the 1980s due to reports of neurological complications [21]. In countries such as the United Kingdom, West Germany, or the United States, the uptake of pertussis immunisation only recovered in the late 1980s and early 1990s [4, 21]. Immunisation was found to be associated with a reduced risk of SIDS in case-control and cohort studies [22, 23]. Reductions in other risk factors for SIDS, such as smoking, could also be observed at the population level [24]. Many risk factors for SIDS are associated with socioeconomic status and tend to cluster in high-risk populations [12, 25, 26].

### Limitations

One limitation of our study was the missing data on SUID and SIDS for certain periods of time in a number of countries. Another limitation is that some of the observed differences may have been caused by artefacts as definitions of SIDS as well as diagnostic procedures varied between countries and over time [2, 27]. The definition of SIDS has changed since its original implementation in 1969, with a stronger focus on death scene investigation including a complete autopsy as requirement for the diagnosis [28]. An increasing reluctance by death certifiers to diagnose SIDS without a thorough investigation might have led to the increase in other diagnoses, as observed in the United States [17]. To our knowledge, there is no systematic assessment of international autopsy rates in infants dying from SIDS in countries over time. In a study comparing eight countries, the estimated percentage of SIDS cases being autopsied differed largely between countries with, for example, particularly low autopsy rates reported for Japan and the Netherlands [14, 16]. The low autopsy rate in Japan might be associated with the observed higher rate of the diagnoses ill-defined and unknown causes of mortality. The comprehensiveness of the autopsy protocol may vary between countries [29]. Often, no systematic information is available on whether an autopsy and/or death-scene investigation was performed according to standard protocols [16]. In general, death certifiers and pathologists may individually or regionally be more likely to over- or underdiagnose SIDS [16]. The age of inclusion as SIDS differed between countries [2]. Some countries defined SIDS as death from 1 week to 12 months, while others used birth to 12 months or beyond. As the majority of SIDS occurs between two and four months, the effect is likely to be minor [12, 30]. The definition of live births similarly varied between countries [8]. However, most countries adopted the standard definition of the WHO in the late 1980s or early 1990s [31]. During the time period of interest, the ICD coding systems changed, which might have impaired comparability over time. The changes in ICD systems, definitions, and coding are less likely to affect the comparability of the aggregate diagnosis of SUID than of SIDS and other individual diagnoses.

## Conclusions

The identification of country clusters in our study may promote research into how changes of risk factors such as smoking, immunisation, or other factors on the population level are related to SUID mortality. Of particular interest are comparisons of time trends between countries with a low - or intermediate - all-cause infant mortality, showing differential levels of SUID and SIDS mortality. While some data on the prevalence of risk factors may already be available, more international collaboration is needed to assess sleep environment and other risk factors in a standardized way for comparison between countries. Compliance with definitions for SIDS and SUID/SUDI will further increase the validity of international comparisons. Innovative methods of statistical analysis and data linkage may be of added value to generate new hypotheses for the prevention of sudden infant death.

## Supplementary information

**Additional file 1.** Distribution of sudden unexpected infant death by its component diagnoses, according to country (1980, 1990, 2000); SIDS (ICD-10, R95), ill-defined and unknown causes of mortality (ICD-10, R96–99), accidental suffocation and strangulation in bed (ICD10, W75)

## Data Availability

The National Statistical Offices of the respective countries provided the data. In the case of missing data, we included additional data from the WHO Mortality Database if available [18].

## Abbreviations

CDC: Centers for Disease Control and Prevention
CI: Confidence interval
GDR: German Democratic Republic
ICD: International Classification of Deaths
SIDS: Sudden infant death syndrome
SUID: Sudden unexpected infant death
USSR: Union of Soviet Socialist Republics
WHO: World Health Organization
