# Supplementary material for: International time trends in sudden unexpected infant death, 1969–2012
Source: BMC Pediatr. 2020 Aug 11;20:377. doi: 10.1186/s12887-020-02271-x (PMC7418369; doi:10.1186/s12887-020-02271-x)
Supplement: Supplementary file 1 — Additional file 1. Distribution of sudden unexpected infant death by its component diagnoses, according to country (1980, 1990, 2000); SIDS (ICD-10, R95), ill-defined and unknown causes of mortality (ICD-10, R96–99), accidental suffocation and strangulation in bed (ICD10, W75) [file 12887_2020_2271_MOESM1_ESM.docx]

1980

1990

2000

Additional file 1: Distribution of sudden unexpected infant death by its component diagnoses, according to country (1980, 1990, 2000); SIDS (ICD-10, R95),
ill-defined and unknown causes of mortality (ICD-10, R96-99), accidental suffocation and strangulation in bed (ICD10, W75)
